# Supplementary material for: Optimized Detoxification of a Live Attenuated Vaccine Strain (SG9R) to Improve Vaccine Strategy against Fowl Typhoid
Source: Vaccines (Basel). 2021 Feb 3;9(2):122. doi: 10.3390/vaccines9020122 (PMC7913755; doi:10.3390/vaccines9020122)
Supplement: Supplementary file 1 [file vaccines-09-00122-s001.pdf]

Supplementary Table S1. Primers used in this study

Primers	5' to 3'	Purpose	Reference
rfaJ deletion F	CTTTAAACGTAAACTTCTTGAATAAAACCCATAGGTGATGTAATGGATTAAATTAACCCTCACTAAAGGGCG	*rfaJ* deletion	This study
rfaJ deletion R	AGTTTTTAATCTTTTTTTCAATAATCATAATAGAGATTTAGGCAGGGGAATAATACGACTCACTATAGGGCTC		
phoP/phoQ deletion F	CAACGCTAGACTGTTCTTATTGTTAACACAAGGGAGAAGAGATGATGCGCAATTAACCCTCACTAAAGGGCG	*phoP/phoQ* deletion	
phoP/phoQ deletion R	ATAACGGATGCTTAACGAGATGCGTGGAAGAACGCACAGAAATGTTTATTTAATACGACTCACTATAGGGCTC		
lpxL deletion F	CAAAAAGATGCGAGAATACGGGGAATTGTTCGTTGAAAGACAGGATAGAAAATTAACCCTCACTAAAGGGCG	*lpxL* deletion	
lpxL deletion R	AAAGCTAAAAGAGGGGAAAAATTGCAGCCTGACGGCTGCAATCCTGTCAATAATACGACTCACTATAGGGCTC		
lpxM deletion F	GACGTCGCTACACTATTCACAATTCCTTTTCGCGTCAGCAGACCCTGGAAAATTAACCCTCACTAAAGGGCG	*lpxM* deletion	
lpxM deletion R	CATCAGGTAGTACAGGGTTTGTCAGCATAAAGCCTCTCTTACGAGAGGCTTAATACGACTCACTATAGGGCTC		
pagP deletion F	TATTCAGGTTAATGTTGTTATTATCACAGTCGAATTTTTGAACGGTATGTAATTAACCCTCACTAAAGGGCG	*pagP* deletion	
pagP deletion R	GGCTTTTTAATTCACAACAGAACAATGCCCTTCTCCGTCAAAACTGGAAATAATACGACTCACTATAGGGCTC		
phoP/phoQ F	CTGTTTATCCCCAAAGCACC	Deletion confirmation	
phoP/phoQ R	GCGAGAGCGGATCAATAAAG		
lpxL F	GCTCAACGCAAAAAGATGCG		
lpxL R	AGGGTGACATAGCGTTCCAC		
lpxM F	CGATTAACAAATGCGCTGAC		
lpxM R	GTTCAACCAATACCACGCGT		
pagP F	CGCCGTTAACCCGATACTCT		
pagP R	GCTGTGTCGGATACCAGTAC		
rfaJ F	TCCAGTCGATGCTGATACTG		
rfaJ R	GTAAACCCTTCTCGCCGAAC		
TNF-α F	CCCCTACCCTGTCCCACAA	qPCR	

TNF-α R	TGAGTACTGCGGAGGGTTCAT	Han et al. (2017)
GAPDH F	CCCCAATGTCTCTGTTGTTGAC	
GAPDH R	CAGCCTTCACTACCCTCTTGAT	
IL-1β F	GCTCTACATGTCGTGTGTGATGAG	
IL-1β R	TGTCGATGTCCCGCATGA	
iNOS F	GCATTCTTATTGGCCCAGGA	
iNOS R	CATAGAGACGCTGCTGCCAG	
IL-18 F	ACGTGGCAGCTTTTGAAGAT	Rychlik et al. (2009)
IL-18 R	GCGGTGGTTTTGTAACAGTG	
TLR-4 F	GGCAAAAAATGGAATCACGA	Rajput et al. (2013)
TLR-4 R	CTGGAGGAAGGCAATCATCA	

Abbreviations: TNF-α, tumor necrosis factor-α; GAPDH, glyceraldehyde 3-phosphate dehydrogenase; IL, interleukin; iNOS, inducible nitric oxide synthase; TLR-4, Toll-like receptor-4.
